# Pressurized Hot Water Extraction of Mangosteen Pericarp and Its Associated Molecular Signatures in Endothelial Cells

**DOI:** 10.3390/antiox12111932

**Published:** 2023-10-30

**Authors:** Sakeena Si Yu Tan, Meyammai Shanmugham, Yu Ling Chin, Jia An, Chee Kai Chua, Eng Shi Ong, Chen Huei Leo

**Affiliations:** 1 Pillar of Engineering Product Development, Singapore University of Technology & Design, Singapore 487372, Singapore; sakeena_tan@mymail.sutd.edu.sg (S.S.Y.T.); cheekai_chua@sutd.edu.sg (C.K.C.); 2 Center for Healthcare Education, Entrepreneurship and Research (CHEERS), Singapore University of Technology & Design, Singapore 487372, Singapore; jia_an@sutd.edu.sg (J.A.); engshi_ong@sutd.edu.sg (E.S.O.); 3 Science, Math & Technology, Singapore University of Technology & Design, Singapore 487372, Singapore; meyammai_shanmugham@mymail.sutd.edu.sg (M.S.); 2000087c@student.tp.edu.sg (Y.L.C.)

**Keywords:** mangosteen pericarp, food waste, PHWE, antioxidant, green extraction, sustainability

## Abstract

The mangosteen (*Garcinia mangostana* L.) pericarp is known to be rich in potent bioactive phytochemical compounds such as xanthones, which possess pharmacologically important antioxidant activity and beneficial cardiometabolic properties. Mangosteen pericarp is typically classified as unavoidable food waste and discarded, despite being rich in bioactive phytochemical compounds that therefore present an exciting opportunity for valorization. Thus, this study aims to extract phytochemical compounds from mangosteen pericarp using pressurized hot water extraction (PHWE) and determine its biological effects in endothelial cells using RNA sequencing. Liquid chromatography with MS/MS (LC/MSMS) and UV detection (LC/UV) was subsequently used to identify three key phytochemical compounds extracted from the mangosteen pericarp: α-Mangostin, γ-Mangostin, and Gartanin. Within the tested range of extraction temperatures by PHWE, our results demonstrated that an extraction temperature of 120 °C yielded the highest concentrations of α-Mangostin, γ-Mangostin, and Gartanin with a concomitant improvement in antioxidant capacity compared to other extraction temperatures. Using global transcriptomic profiling and bioinformatic analysis, the treatment of endothelial cells with mangosteen pericarp extracts (120 °C PHWE) for 48 h caused 408 genes to be differentially expressed. Furthermore, our results demonstrated that key biological processes related to “steroid biosynthesis and metabolism”, likely involving the activation of the AMPK signaling pathway, were upregulated by mangosteen pericarp extract treatment. In conclusion, our study suggests a green extraction method to valorize phytochemical compounds from mangosteen pericarp as a natural product with potential beneficial effects on cardiometabolic health.

## 1. Introduction

A tropical fruit native to Southeast Asia, the mangosteen (*Garcinia mangostana* L.) belongs to the Clusiaceae family. With its distinctive sweet–sour flavor and various therapeutic applications, the fruit is referred to as the “queen of tropical fruits” [1]. The mangosteen fruit is reddish/dark purple with a luscious, soft, edible pulp and exquisite taste. The fruit’s pericarp comprises three layers: an innermost endocarp layer, a fleshy mesocarp layer in the center, and an exocarp layer that is thick, rigid, and inedible on the outside [2,3]. Bioactive phytochemical substances found in the fruit include xanthones (e.g., α-Mangostin), procyanidins, flavonoids, and anthocyanins, which are mostly present in the pericarp [2,3]. These bioactive phytochemical substances have been linked to several beneficial biological properties, including antioxidant, antibacterial, antidiabetic, antiproliferative, and anticancer properties [1,2,3]. Given that oxidative stress and inflammation are known to be commonly associated with several chronic diseases such as cardiovascular diseases, cancer, and metabolic diseases [4,5,6,7,8], previous studies have demonstrated a highly enriched concentration of α-Mangostin in mangosteen pericarp extract, possessing antioxidant, anticancer, and cytotoxic properties [3,9,10,11]. Considering how the pericarp constitutes a large fraction of the fruit and is rich in bioactive phytochemicals, coupled with the fact that the pericarp is generally unconsumed and considered as unavoidable waste, this presents an opportunity to develop suitable extraction methods to upcycle these bioactive phytochemicals into functional food products suitable for beneficial health-related applications.

The concentration and polarity of the extraction solvents have been found to be fundamental variables in the crucial process of extracting phytochemicals from the mangosteen pericarp [12]. Various organic solvents, such methanol, ethanol, and acetone, are frequently used to extract a variety of secondary metabolites from plant sources [13,14]. Depending on the plant species and the chemicals being extracted, these solvents may be used. However, the use of organic solvents is generally not environmentally friendly or sustainable and may also result in increased costs of production as the resultant organic wastes would require proper disposal [15,16]. In addition, phytochemicals extracted using organic solvents would require additional purification steps to eliminate these solvents before they are safe for consumption, which complicates the overall extraction process to obtain the required phytochemicals from the material [15,16]. With the recent global interest in green technologies and sustainability [17,18], several studies have utilized green extraction principles to valorize phytochemical extracts from various plants sources, including mangosteen pericarp [14,19]. Green extraction techniques are known to consume less energy, allow for the use of safe alternative solvents (non-organic), and utilize renewable natural resources [20,21,22]. Some of these green extraction techniques include microwave assisted extraction, deep eutectic solvent extraction and pressurized hot water extraction (PHWE) [19,23,24]. Although these methods are typically regarded as “greener” than conventional ones, they may also need unique instruments and intricate procedures, which may impact their extraction efficiency, extraction yield, and energy usage [18,19]. Recently, the advantages and disadvantages of using PHWE as a green extraction method has been extensively reviewed [19]. In the case of PHWE, water is heated to temperatures between 100 and 374 °C under a constant pressure and employed as the extraction solvent. This results in a decrease in the dielectric constant linked to the polarity of water, allowing for the extraction of less polar chemicals and increasing extraction efficiency [19,25,26]. The extraction procedure with PHWE is less complex as it does not require an additional solvent removal step. Moreover, PHWE may be more energy-efficient than other methods since it does not require heating and cooling systems that are both functioning simultaneously. Therefore, the extraction process of PHWE is simple and rapid, and the process can be scaled up when needed. Furthermore, it enables the recovery of non-polar compounds even with the complete elimination of organic solvent, which is environmentally friendly [19,25,26]. Thus, PHWE is only one of the green extraction methods listed in the Compendium of Terminology in Analytical Chemistry [27]. As PHWE involves high temperatures, the possible disadvantage is the degradation of bioactive compounds; hence, the characterization of the biological and cellular effects of the extracts will be critical to ensure the viability of the bioactivity compounds [19].

As green extraction methods are more environmentally friendly to organic solvents, some of the earlier studies have determined the extraction yield and efficiency of phytochemicals (e.g., α-Mangostin) extracted from the mangosteen pericarp [28,29]. However, the effect of these extracts on the cellular targets and their associated molecular signatures remains unclear in endothelial cells, which are important regulators of vascular homeostasis [30,31]. Thus, the aim of the present study is to use PHWE to extract phytochemical compounds from mangosteen pericarp and determine the biological effects of mangosteen pericarp extract (MPE) in endothelial cells. Specifically, using RNA sequencing, the transcriptomic profile and pathway enrichment analysis of the cellular effects of MPE in endothelial cells will be characterized.

## 2. Materials and Methods

### 2.1. Sample Preparation and Chemicals 

HPLC-grade water, formic acid, methanol, sand, 2,2′-Azinobis-3-ethylbenzothiazoline-6-sulfonic acid (ABTS), 1,1-diphenyl-2-picrylhydrazyl (DPPH), and standards used in LC/UV were purchased from Sigma-Aldrich (Singapore). Mangosteen pericarps were lyophilized and blended using a food blender into powder form and sieved to obtain a powder with particle sizes of 0.3 < 1.0 mm. The 0.3 < 1.0 mm powder was used for the respective extraction methods. 

### 2.2. Extraction of Compounds Using PHWE 

To extract and analyze the bioactive compounds within MPE, a PHWE system was used. Specifically, the laboratory PHWE system included an isocratic Shimadzu LC10 series pump (Kyoto, Japan), a stainless-steel extraction cell (250 × 10 mm i.d.), stainless-steel tubing of 1/16 inches o.d. and 0.18 mm i.d. for connections, and a Hewlett Packard 5890 Series II DVS 402 constant temperature oven to ensure efficient heat transfer [26]. Mangosteen pericarp samples were weighed to 0.75 g in 50 mL tubes and mixed with a small proportion of sand; then, they were vortexed with a benchtop vortex for homogeneity and to maintain back pressure during the extraction process. The samples were then loaded into the extraction cell and tightened into the PHWE system via the connecting tubes. The flow rate of the pump was set at a constant rate of 1.2 mL/min for 40–50 min. The extraction processes were repeated in triplicate, with 45 mL of extracts collected during each repeat across four different temperatures each: 60 °C, 80 °C, 100 °C, and 120 °C. All triplicates for each temperature were subsequently combined and concentrated, freeze-dried, and stored for future reconstitution in subsequent experiments. 

### 2.3. LC/UV and LC/MSMS Profiling of Mangosteen Pericarp Extracts

Using LC/UV, the chemical profiles of each mangosteen pericarp were identified. In order to assess the phytochemical characteristic profiles in the MPE, the obtained extracts were filtered through a 0.2 µm filter into vials and set up on the auto-sampling rack. A C18 reverse-phase HPLC column (Zorbax SB-C18 3.5 microns, 4.6 × 100 mm; Agilent, Santa Clara, CA, USA) was used to separate the chemicals [26]. The column temperature was kept at 40 °C, the total flow rate was set at 0.8 mL/min, and the injection volume was supplied to the system at 10 µL and simultaneously separated into the HPLC (Shimadzu Nexera X-2, Kyoto, Japan) apparatus. The mobile phase for the gradient elution included 0.1% formic acid in (A) water and (B) acetonitrile, respectively. The UV detector identified the presence of bioactive substances based on the standards (-Mangostin, -Mangostin, and Gartanin) at the absorbance value of 254 nm. The calibration curve, relative standard deviation (RSD), and coefficient of determination *r*^2^ for LC/UV were determined. All three standards utilized had *r*^2^ values of at least 0.9990, showing a significant correlation between the calculated and real values. RSD values (%) were ≤1.00% for eight consecutive injections of the individual analytes, demonstrating the low variation and strong repeatability of findings (Supplementary Table S1). Prior to obtaining an average reading for the three samples, the recorded peak intensities for each sample were normalized to make the peak areas a consistent total. Peaks for LC/UV were identified with wavelengths at 254 nm. A principal component analysis (PCA) score plot was used to further analyze the normalized peak regions for each sample. The present methodology is consistent with our prior studies [26,32]. 

For LC/MSMS, a C18 reverse-phase HPLC column (Zorbax SB-C18 3.5 microns, 2.1 X 50 mm; Agilent, USA) was used. The total flow rate was set at 0.3 mL/min and the column temperature was set at 40 °C. The injection volume was 2 µL. The LC/MSMS used were the Nexera X-2 HPLC (Shimadzu, Japan) and LC/MS-8050 (Shimadzu, Japan) machines. The gradient elution consisted of a mobile phase involving 0.1% formic acid in (A) water and (B) methanol, respectively. The gradient parameter was performed in a negative mode, for 0.10 min at 5%, 10.00 min at 100%, 10.10 min at 5% and 15.10 min at 5%; this was to re-equilibrate the column back to the initial conditions. The electrospray ionization (ESI) was carried out with a nebulizing gas flow of 2.8 L/min, interface temperature of 300 °C, and a heating and drying gas flow of 9.0 L/min. The standards and MPE were filtered through a 0.45 µm filter into respective vials and placed into the auto-sampling rack for compound analysis. 

### 2.4. DPPH (1,1-Diphenyl-2-picrylhydrazyl) Antioxidant Assay

The antioxidant activity of the mangosteen pericarp extracts was tested by DPPH (1,1-diphenyl-2-picrylhydrazyl) and ABTS (Azinobis-3-ethylbenzothiazoline-6-sulfonic acid) colorimetric assays [26,32]. The antioxidant activity of the mangosteen pericarp was tested using the DPPH assay at PHWE temperatures of 60 °C, 80 °C, 100 °C, and 120 °C, as previously discussed [26,32]. Briefly, a 96-well plate was filled with 100 µL of each of the MPE extracts (5 µg/mL–0.1 mg/mL) or the negative control (ddH_2_O). Subsequently, 100 µL of a homogeneously mixed 0.1 mM DPPH solution was pipetted into those wells. For the positive control, 10 mM ascorbic acid was used in place of the MPE. The Multiskan GO microplate reader from Thermo Scientific (Singapore) was used to measure the absorbance at 517 nm after incubation. Readings were processed as below:

$$\%\;\mathrm{maximum}\;\mathrm{inhibition}=\frac{A_{n}-A_{s}}{A_{n}-A_{p}}\times 100$$

where A_n_ refers to the negative control absorbance value, A_s_ refers to the sample absorbance measured and A_p_ refers to the absorbance measured from the ascorbic acid. All experiments were performed in triplicate.

### 2.5. ABTS (Azinobis-3-ethylbenzothiazoline-6-sulfonic Acid) Antioxidant Capacity Assay

The vitamin C equivalent antioxidant capacity (CEAC) values were determined using an ABTS assay as previously described [26,33]. The 7 mM ABTS solution was combined with 88 µL of a 140 mM potassium persulfate solution for the ABTS test, and the resultant solution was left to incubate at 4 °C for 16 h in the dark. The final product of the incubation procedure was a dark purple solution made of ABTS^•+^ that was then diluted with around 42.5 mL of methanol to produce an initial absorbance of 0.70 to 0.80 at 734 nm [26,33]. A standard curve for ascorbic acid was created (100 μM to 1 mM) to induce a 5–50% suppression of the blank absorbance (ABTS^•+^ alone) [26,33]. Subsequently, 25 μL of the MPE sample extracts from the various temperatures (60 °C, 80 °C, 100 °C, and 120 °C) was then added to the 96-well microplate that already contained 200 μL of the ABTS^•+^ solution. The Multiskan GO microplate reader (Thermo Scientific, Singapore) was used to detect absorbance at 734 nm after the reaction had been incubated for 30 min at 25 °C with low-frequency shaking. All experiments were performed in triplicate [26,33]. Briefly, the ascorbic acid standard curve that was created resulted in a 5 to 50% scavenging of pre-formed radicals. The equation derived from the linear regression of the standard curve was used to determine the CEAC values for the MPE samples [26,33]. 

### 2.6. Cell Culture and Treatment with MPE

Human Dermal Microvascular Endothelium (HMEC-1) cells were obtained (American Type Culture Collection [ATCC], Manassas, VA, USA) and cultured in MCBD-131 media (20% fetal bovine serum (FBS), 5% L-glutamic acid (200 mM), 1% penicillin-streptomycin and 0.001% recombinant human epidermal growth factor (EGF) (10 ng/mL)) using T75 flasks at 37 °C with 5% CO_2_. Cells were seeded into cell culture plates at 75–85% confluency under serum-starved conditions (2% FBS) and incubated for 24 h [25]. The HMEC-1 cells were damaged with trimethylamine N-oxide (50 µM) for 48 h to induce endothelial dysfunction [34] in the absence (control) or presence of MPE (0.05 mg/mL). After treatment, the cells were harvested for RNA sequencing experiments. 

### 2.7. RNA Extraction and RNA Sequencing

After 48 h of treatment, the wells were washed with phosphate-buffered saline (PBS) and RNA was extracted using the Aurum^TM^ Total RNA Mini Kit (Bio-Rad, Hercules, CA, USA), and the RNA quality and quantity were analyzed as described earlier [33]. The total RNA from the treated HMEC-1 was used to construct libraries using the Illumina HiSeq platform PE150 at Azenta Life Sciences (Hangzhou, China). Bioinformatics pipeline analysis was performed using the raw FASTQ reads from the RNA sequencing data, which was processed using the Galaxy platform following the industry standard nf-core pipeline v-3.6 [35]. Sequencing and read quality were examined using QC stats tools. Genome annotation and quantification were performed using HiSAT2 and feature count tools [35]. 

### 2.8. Transcriptional Profiling and Regulatory Network Analysis

Differentially expressed genes were generated using the Limma Voom tool (version 3.50.1). Pair-wise comparisons were performed for the treatment group (MPE) vs. the control. A log fold-change threshold of ±0.4 was set as an arbitrary value with an FDR *p*-value < 0.05 for the differential gene expression selection. A graphical tool for gene-set enrichment analysis, ShinyGo, was used to analyze the top differentially expressed genes in both upregulated and downregulated processes [36]. Enriched gene ontology (GO) biological processes, function, networks, and the Kyoto Encyclopedia of Genes and Genomes (KEGG) pathway were analyzed [37,38,39], and their diagrams were obtained [36]. GO biological processes and KEGG pathways were screened at a *p*-value < 0.05.

### 2.9. Statistical Analysis

The Soft Independent Modelling by Class Analogy (SIMCA) software (Version 16) produced PCA charts. A sigmoidal curve was produced by fitting concentration–response curves for DPPH inhibition using GraphPad Prism 6 software (GraphPad, San Diego, CA, USA). To determine the MPE’s sensitivity to DPPH inhibition (IC_50_), nonlinear regression was used. One-way ANOVA was used to analyze group mean values, and Tukey’s test was used for post-hoc analysis. All data are presented as mean ± SEM. Statistical significance was considered when the *p*-value was <0.05.

## 3. Results and Discussion

### 3.1. PHWE and Chemical Standardization of MPE

**Figure 1:**
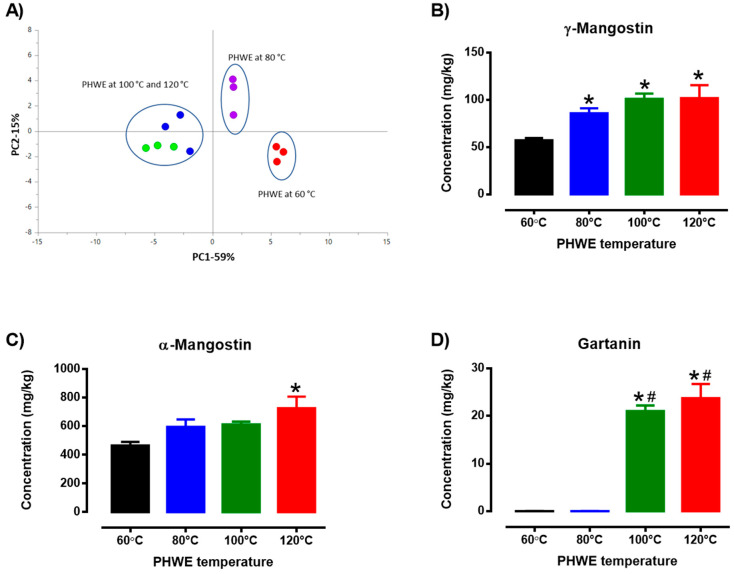
(**A**) PCA score plot (PC1 X PC2) of LC-UV profile at UV 254 nm of MPE obtained from the different PHWE temperatures (60 °C, 80 °C, 100 °C, 120 °C). LC/UV analysis of the amount of standard compounds, (**B**) γ-Mangostin, (**C**) α-Mangostin, and (**D**) Gartanin extracted from different PHWE temperatures. Each bar is represented by mean ± SEM, n = 5–6. * Significantly different to 60 °C extraction temperature, # Significantly different to 80 °C extraction temperature, *p* < 0.05, one-way ANOVA, Tukey’s post-hoc test.

It has been well established that the extraction efficiency of PHWE is dependent on several factors such as flow rate, applied temperature, solvent system and particle size. However, the applied temperature is a critical factor that has been observed to affect the yield of each compound [23]. For the current work, three marker compounds—α-Mangostin, γ-Mangostin and Gartanin—were selected to evaluate the extraction efficiency. Specifically, LC/MSMS was performed to identify the presence of α-Mangostin, γ-Mangostin and Gartanin from MPE with reference to the MSMS spectra obtained from the reference standards (Table 1, Supplementary Figure S1), which is also consistent with a recent report [40]. Subsequently, concentrations of the target compounds were determined at the various applied temperatures based on external calibration via reverse calculation using the peak areas and standard curves produced during the LC/UV methodology, as previously mentioned [41]. Based on target compounds and peaks detected using LC/UV, PHWE extraction at different temperatures yielded MPEs with unique chemical profiles (Figure 1A). Specifically, it is evident that different PHWE temperatures probably produce a distinct chemical fingerprint as there was a clear separation among the three clusters: PHWE temperatures at 60 °C, 80 °C, and higher (100 °C and 120 °C), suggesting that increasing the temperature of PHWE causes the extraction of different phytochemical compounds of different compositions. The present method of chemical fingerprinting and observations were comparable with our past studies in which a distinctive chemical fingerprint and the quantity of various target chemicals could be found in samples extracted using different extraction methods [25,26,32,33]. Therefore, these chemical profiles suggest that the extraction of mangosteen pericarp using PHWE would probably produce a distinct chemical fingerprint and concentration of the target chemicals based on different extraction temperatures (Figure 1A).

Mangosteen pericarp is known to contain phytochemicals such as α-Mangostin, γ-Mangostin and Gartanin [42]. In this study, the amount of α-Mangostin, γ-Mangostin and Gartanin were determined, which was consistent with previous studies [9]. However, different extraction temperatures using PHWE led to various concentrations of the identified chemical yields (Figure 1B–D). Specifically, in this study, there was a temperature-dependent effect on the yield of γ-Mangostin (Figure 1B) and α-Mangostin (Figure 1C), which was significantly higher with increasing extraction temperatures (60 °C to 120 °C). It was observed that the concentration of γ-Mangostin was the lowest at 60 °C PHWE, and the yield of γ-Mangostin was significantly increased with increasing temperatures (80 to 120 °C) when compared to 60 °C PHWE (Figure 1B). While the concentration of α-Mangostin was comparable between the lower temperatures (60 °C to 100 °C) of PHWE, it was found to yield the highest concentration at 120 °C PHWE and was significantly increased when compared to 60 °C PHWE (Figure 1C). Based on Figure 1D, the applied temperature at 100 °C and 120 °C PHWE was found to yield the highest amount of Gartanin, which was observed to be undetected in the lower temperatures of PHWE (60 to 80 °C). 

Most phytochemicals, including α-Mangostin, are natural organic compounds with low polarity and may be successfully extracted using water as an extraction solvent. Even though water is a polar solvent, raising the temperature of extraction under higher pressure conditions would lower its dielectric constant and reduce the polarity of water, which would cause the extraction of non-polar chemicals [23]. Indeed, higher PHWE temperatures have probably contributed to higher α-Mangostin yields when comparing extraction at a lower temperature of 60 °C. Furthermore, previous studies have shown that increasing the extraction temperature during subcritical water extraction resulted in increased amounts of α-Mangostin extracted from the mangosteen pericarp [9,42]. Despite the differences in the overall known (α-Mangostin, γ-Mangostin and Gartanin) and unknown chemicals extracted using PHWE, the antioxidant properties of the MPE will be evaluated. 

### 3.2. Antioxidant Activity of MPE

**Figure 2:**
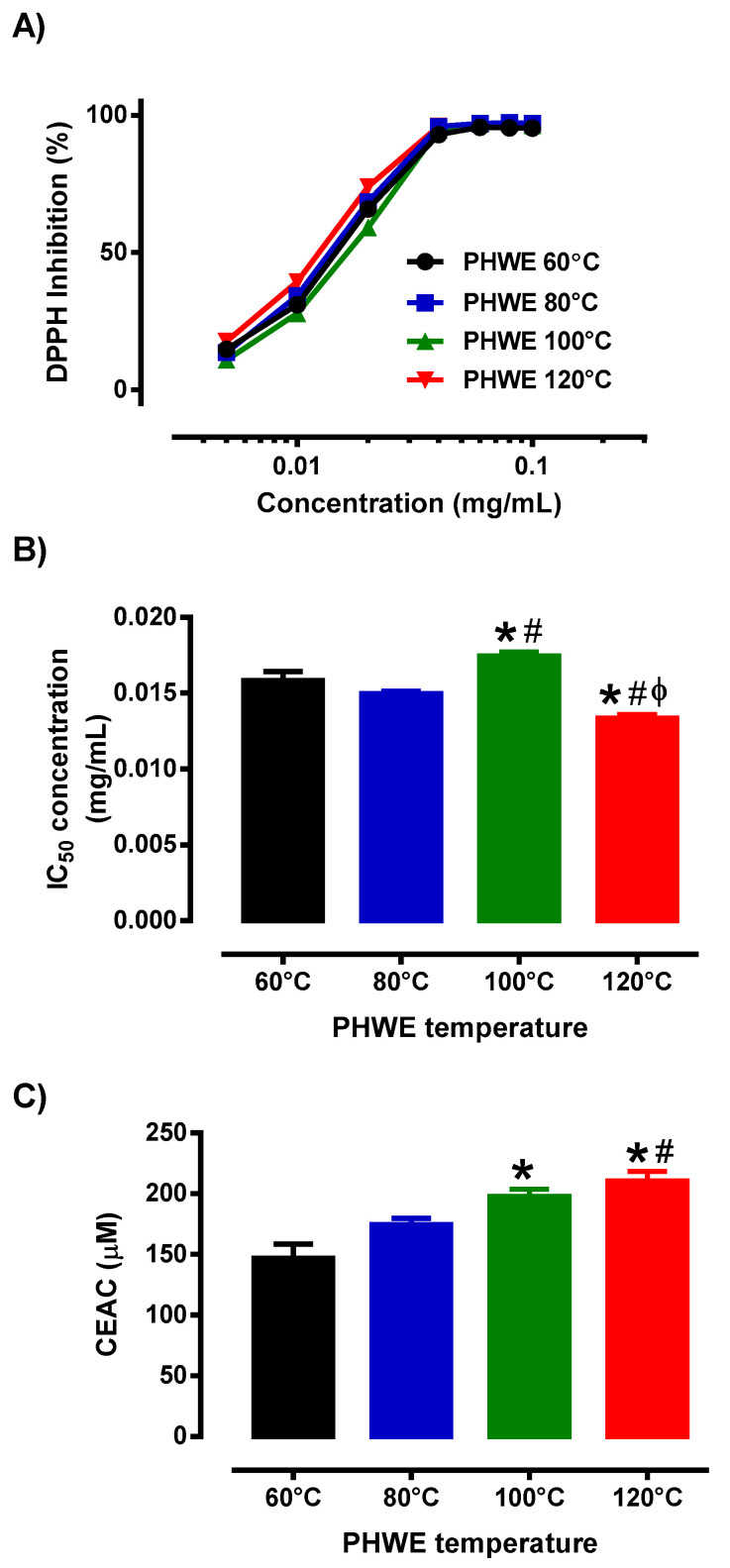
Antioxidant activity of MPE obtained from different extraction conditions was evaluated using two different assays, (**A**,**B**) DPPH and (**C**) ABTS. (**A**) Concentration response curve of MPE and (**B**) inhibitory concentration (IC_50_) value and (**C**) CEAC of MPE (0.1 mg/mL) obtained from different PHWE temperature. Each bar is represented by mean ± SEM, n = 3–4. * Significantly different to 60 °C extraction temperature, # Significantly different to 80 °C extraction temperature, Φ Significantly different to 100 °C extraction temperature, *p* < 0.05, one-way ANOVA, Tukey’s post-hoc test.

Given the differential chemical profile (Figure 1A), it is unclear if the variable extraction condition has any impact on the antioxidant activity of MPE, a well-reported biological property of polyphenolic phytochemicals. Hence, by using two separate antioxidant protocols, we assessed the antioxidant potential of the MPE at various PHWE temperatures. Based on the DPPH and ATBS experiments, MPE obtained at various PHWE temperatures demonstrated antioxidant activity, where increasing concentrations of the MPE caused increased scavenging of the free radical, DPPH (Figure 2A). These findings were consistent with earlier studies [9,13,28,43]. Specifically, in addition to direct radical scavenging, mangosteen pericarp has been shown to have protective effects against oxidative stress by regulating the activities of several key endogenous, including superoxide dismutase (SOD), glutathione peroxidase (GPx) and catalase (CAT) through the nuclear factor erythroid 2-related factor 2 (Nrf2) transcription factor, which targets genes involved in antioxidant, detoxification, metabolism, and inflammatory pathways [44]. Furthermore, using a mouse light-damage model to increase the production of H_2_O_2_, which activates caspase 3, leads to retinal death [43]. However, mangostin (30 mg/kg) treatment boosted Nrf2 translocation to the nucleus after light exposure, promoting the production of antioxidant genes, lowering the expression of cleaved caspase-3, and preventing retinal damage. Similarly, the supplementation of a 200–600 mg/kg dose of mangosteen extract reduced high-fed diet-induced oxidative stress conditions by improving SOD and GPx levels in an obese rat model [10]. 

While there are well-documented antioxidant effects of mangosteen pericarp from in-vitro and in-vivo studies [9,13,28,43], in this study, we demonstrated that different PHWE temperature extractions of mangosteen pericarp exhibited distinct antioxidant efficacy. Specifically, the MPE from the higher temperatures (100 °C to 120 °C) of PHWE reported small but significant differences in IC_50_ concentrations when compared to MPE obtained from PHWE at the lower temperatures (60 °C to 80 °C). Furthermore, the reported IC_50_ concentration of MPE obtained from PHWE at 120 °C was significantly lower than the other PHWE temperatures, indicating that the potency of the antioxidant activity was highest in MPE obtained from PHWE at a 120 °C extraction temperature (Figure 2B). Furthermore, our results from ABTS experiments demonstrated that CEAC for PHWE at 100 °C and 120 °C were significantly higher compared to other PHWE temperatures (60 °C and 80 °C) (Figure 2C). One possible explanation for the observed small, yet significant, differences in antioxidant capacity may be attributed to the unique composition of polyphenolic phytochemicals present in the MPE. For example, in this study, PHWE at 100 °C and 120 °C yielded Gartanin, which is not present in other conditions and is accompanied by higher antioxidant capacity. Previous structure-activity relationship studies suggested that hydroxyl groups within polyphenolic compounds play an important role in radical scavenging effects and the number of hydroxyl groups within the compound may affect its overall antioxidant capacity [45,46]. Given that Gartanin has more hydroxyl groups compared to α-Mangostin, it is proposed that Gartanin is likely to possess higher antioxidant potency compared to other phenolic acids such as α-Mangostin that were present in the MPE. Collectively, based on the yield and profile of the various phenolic compounds and antioxidant capacity, it is still not entirely certain which polyphenolic phytochemicals in the MPE are responsible for the antioxidant effects. While it has been found that polar compounds extracted from mangosteen pericarp contain higher antioxidant capacities compared to their non-polar counterparts, there might also be other polyphenolic phytochemicals in the MPE that may also have contributed to antioxidant activity that we were unable to identify in this study [47]. 

It is important to note that while the yield of the chemical composition of the mangosteen pericarp may vary using different extraction methods, however, in most cases, the bioactivity of the extracts such as antioxidant capacity is consistent across the different extraction methods. For example, previous studies reported that methanol extracts of dried mangosteen pericarp showed antioxidant activity with an IC_50_ value of 14.7 µg/mL [48] or 20.64 µg/mL [28] compared to the current study (IC_50_ = ~12 µg/mL) using the DPPH assay. Furthermore, the maximum inhibition of DPPH was observed using mangosteen pericarp extracts in these studies [28,48]. Therefore, considering the different varieties of mangosteen used in various studies, the functional components of mangosteen pericarp extracted using PHWE appear to be consistent with other extraction methods [28,48,49]. Based on our previous studies [25,26,33], we observe that distinctive clusters from the PCA score plots from LC/UV were consistent with LCMSMS, suggesting that the unique chemical fingerprint obtained by LC/UV was consistent with that obtained by LCMS. Despite not being able to detect all the polyphenolic phytochemicals present in the MPE, our experiments established that the PHWE condition at 120 °C allowed MPE to exhibit the best antioxidant capacity; this will be used for subsequent experiments to determine the transcriptomics and molecular signatures associated with MPE treatment in endothelial cells. While this study is not able to identify all the chemical components present in MPE, it is important to note that the observed antioxidant activity and associated molecular signatures are likely to be attributed to the full spectrum of MPE, rather than the identified target compounds: α-Mangostin, γ-Mangostin and Gartanin.

### 3.3. Transcriptomic Profiling and Molecular Signatures of MPE Treatment in Endothelial Cells

**Figure 3:**
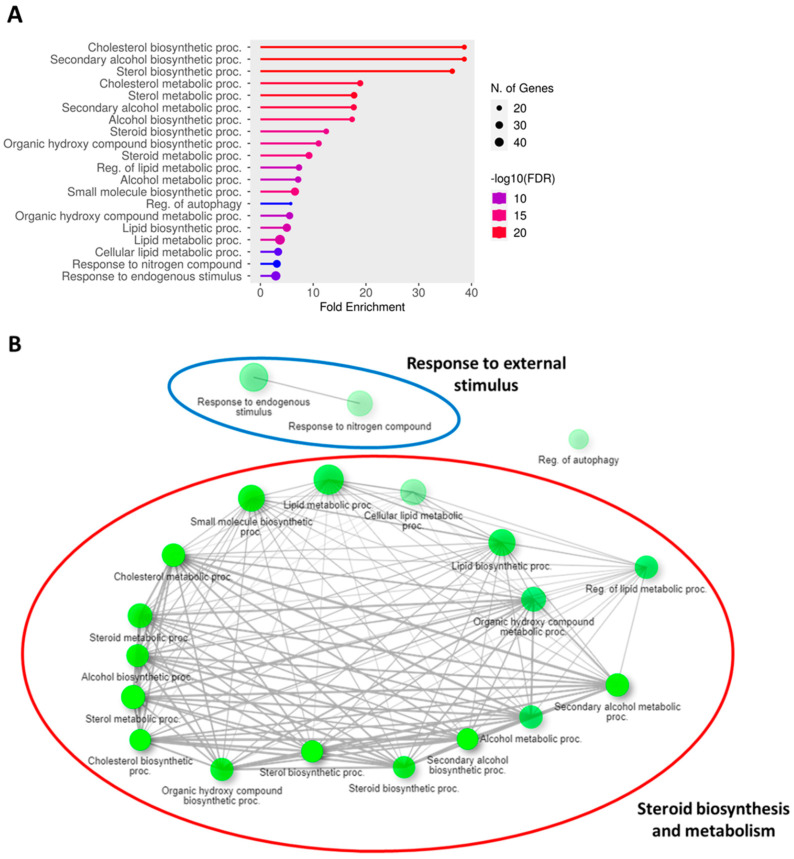
GO biological processes for the DEGs from transcriptomics profiling of HMEC-1 treated with MPE (0.05 mg/mL) for 48 h. (**A**) Enrichment Plot of the GO BPs that correspond to respective upregulated genes. Significant biological clusters were associated with “response to external stimulus” (blue), and “steroid biosynthesis and metabolism” (red). (**B**) Dot plot showing the number of significant genes upregulated by MPE at 48 h. The *x*-axis represents the fold enrichment, and the GO BPs are on the *y*-axis. The number of genes is indicated by the size of the circle and the −log10(FDR) is represented in different colors. DEG was identified using adjusted *p*-value < 0.05 and log_2_FC ≥ = |0.4| criteria, n = 3.

**Figure 4:**
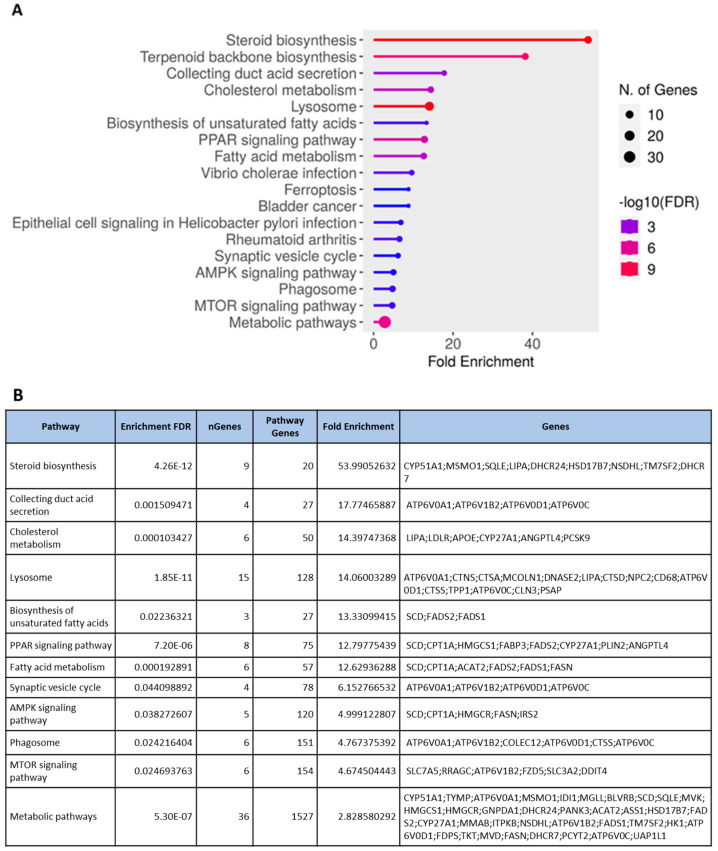
KEGG pathways for the DEG from the transcriptomics profiling of HMEC-1 treated with MPE (0.05 mg/mL) for 48 h. (**A**) Dot plot showing the DEGs upregulated in the respective KEGG pathways by MPE (0.05 mg/mL) at 48 h. The *x*-axis represents the fold enrichment, and the KEGG pathways are on the *y*-axis. The number of genes is indicated by the size of the circle and the −log10(FDR) is represented in different colors. DEG was identified using adjusted *p*-value < 0.05 and log_2_FC ≥ = |0.4| criteria, n = 3. (**B**) Upregulated DEGs corresponding to KEGG pathways are shown. The enrichment FDR, number of genes involved in the pathway, fold enrichment, KEGG pathway, and the genes found in the pathway are represented.

**Figure 5:**
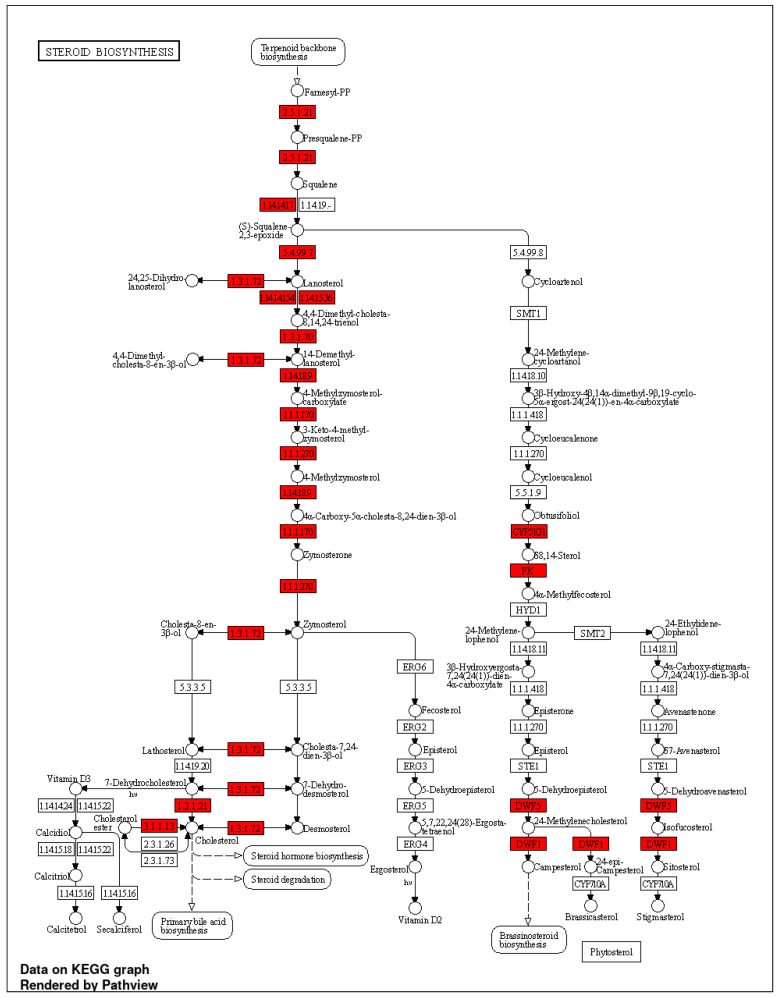
“Steroid biosynthesis” was an upregulated KEGG pathway for the transcriptomics profiling of HMEC-1 treated with MPE (0.05 mg/mL) for 48 h. Gene hits are highlighted in red (upregulated). DEG was identified using adjusted *p*-value < 0.05 and log_2_FC ≥ = |0.4| criteria, n = 3.

Given that PHWE involves high temperatures, a possible disadvantage is the degradation of bioactive compounds; hence, the characterization of the cellular effects of the extracts will be critical to ensure the viability of the bioactivity compounds. Using the global transcriptomic approach, a total of 408 genes were differentially expressed after 48 h of MPE (0.05 mg/mL) treatment, which was based on an adjusted *p*-value < 0.05 and a log_2_FC ≥ = |0.4| threshold. From a total of 408 genes that were differentially expressed, 205 genes were found to be significantly downregulated, while 203 genes were significantly upregulated (Supplementary Figure S2). To have a better biological representation of the differentially expressed genes (DEGs), the list of DEGs was used to perform overrepresentation enrichment analysis using gene ontology biological processes (GO BPs) and the Kyoto Encyclopedia of Genes and Genomes (KEGGs) database to identify key molecular signatures and pathways that were modulated by MPE treatment. MPE treatment caused the significant upregulation of DEGs that corresponded to biological processes associated with “metabolic and biosynthesis processes” mainly for lipid mediators, such as cholesterol and sterol biosynthesis, and “response to endogenous stimulus” (Figure 3A, Supplementary Figure S3). Overall, these key biological processes can be classified into two main clusters, namely, “response to external stimulus” and “steroid biosynthesis and metabolism” (Figure 3B, Supplementary Figure S3). Furthermore, several upregulated biological processes (e.g., “steroid biosynthesis and metabolism” and “cholesterol biosynthesis and metabolism”) correspond to KEGG pathways (Figure 4A,B). Specifically, “steroid biosynthesis” and “cholesterol metabolism” KEGG pathways were also identified to be significantly upregulated pathways after MPE treatment when compared to the control (Figure 4B). Specifically, our study identified the significant enrichment of the “steroid biosynthesis” KEGG pathway, where nine key genes within the pathway were upregulated by MPE treatment (Figure 5). Interestingly, the downregulated DEGs correspond to biological processes such as “positive regulation of gene expression”, and “DNA replication-independent nucleosome assembly” (Supplementary Figure S4). Notably, while 205 genes were significantly downregulated, the enrichment of the biological processes failed to reach statistical significance as they did not meet our adjusted *p*-value < 0.05 threshold, and moreover, no reported KEGGs pathways were associated in our enrichment analysis with downregulated DEGs (Supplementary Figure S4). Therefore, our results demonstrated that MPE treatment has significant overall molecular effects on metabolic processes, particularly on lipid and steroid biosynthesis, suggesting that MPE may have potentially beneficial effects against metabolic syndrome and/or cardiometabolic diseases.

**Figure 6:**
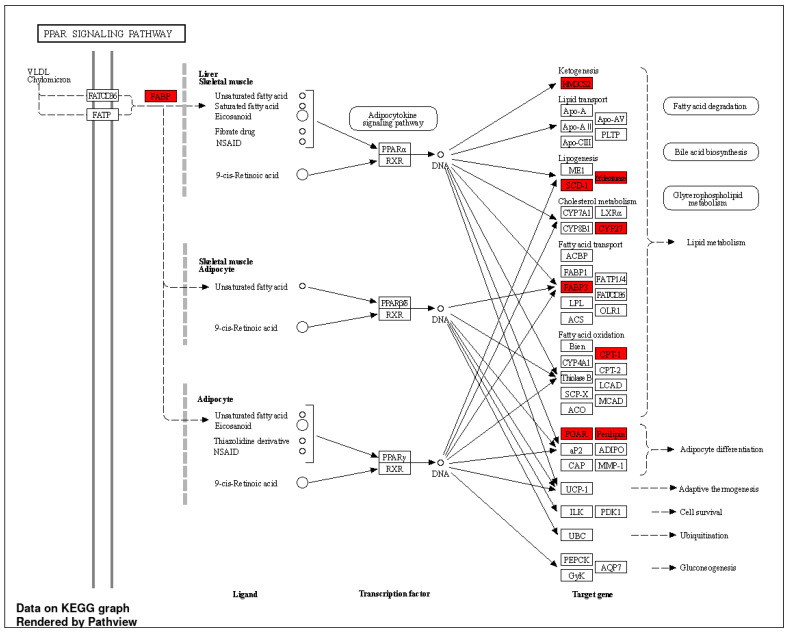
“PPAR signaling pathway” was an upregulated KEGG pathway for the transcriptomics profiling of HMEC-1 treated with MPE (0.05 mg/mL) for 48 h. Gene hits are highlighted in red (upregulated). DEG was identified using adjusted *p*-value < 0.05 and log_2_FC ≥ = |0.4| criteria, n = 3.

The common features of metabolic syndrome include abdominal obesity, atherogenic dyslipidemia, hypertension, and insulin resistance accompanied by hyperglycemia, which contributes to oxidative stress, a pro-inflammatory and prothrombotic condition [31,50]. Cardiovascular disease, type 2 diabetes, and fatty liver are some of the comorbidities linked to the metabolic syndrome [50]. Indeed, mangosteen pericarp extracts have been previously demonstrated to show beneficial effects against metabolic syndrome. According to a study by John and colleagues [51], rats on a high-fat/-carbohydrate diet were shown to experience less body weight growth, visceral fat accumulation, and plasma triglyceride levels when treated with mangosteen rind extract. Similarly, the administration of 200 and 500 mg/kg mangosteen extract per day caused a reduction in body weight gain, which was connected to a reduction in fatty acid synthase activity in adipose tissue and serum [52]. Furthermore, in high-fat-diet C57BL/6 mice, daily doses of 50 and 200 mg/kg of mangosteen extract reduced body weight and visceral fat and improved lipid metabolism by lowering levels of triglycerides and total cholesterols [53,54]. Subsequent mechanistic studies revealed that mangosteen extract treatment promoted body weight loss by increasing the expression of hepatic peroxisome proliferator-activated receptor γ (PPARγ), sirtuin (SIRT) 1, AMP-activated protein kinase (AMPK) and retinoid-X-receptor alpha, suggesting that the anti-obesity effects of mangosteen extract are mediated via the SIRT1-AMPK and PPARγ pathways in mice with obesity induced by a high-fat diet [53,54]. These observations were also consistent with our study as KEGG pathways such as the “PPAR signaling pathway” (Figure 6) and “AMPK signaling pathway” (Supplementary Figure S5) were also significantly upregulated by MPE treatment. It has been well established that AMPK activity preserves cellular energy storage by “switching off” ATP using anabolic pathways and activating catabolic pathways that do so, principally through enhancing oxidative metabolism and mitochondrial biogenesis [55]. Besides metabolic effects, the AMPK pathway is known to possess cardioprotective effects in endothelial cells. A previous study demonstrated that endothelial cells cultured in high glucose (60 mM) drastically decreased cellular viability, increased ROS, and accelerated cellular senescence by reducing the activity of the enzyme linked with cellular senescence [56]. A high-glucose environment significantly increased the amount of IL-6, p53, acetyl-p53, and p21 protein secretions; however, it decreased the levels of SIRT1 and total AMPK proteins. Notably, α-Mangostin (1.25 mM) treatment increased SIRT1 expression while decreasing apoptosis, ROS, and IL-6 production in high-glucose exposed endothelial cells, suggesting that the protective benefits of α-Mangostin to endothelial cells are likely attributed to its antioxidant activity through the SIRT1 pathway [56]. Taken together, the reported beneficial cardiometabolic effects of mangosteen extracts (α-Mangostin) were associated with a reduction in adipose mass, fat accumulation and fatty acid synthase activity through the increased activation of the AMPK-SIRT1 pathways and the increased fatty acid oxidation via increased CPT1A expression [53,54]. Given that the key pathways associated were the AMPK-SIRT1 pathway and the PPARγ pathway, the activation of these pathways may also contribute to the cardioprotective effects of mangosteen extracts in endothelial cells. 

## 4. Conclusions

In conclusion, mangosteen pericarp consists of a rich source of natural phytochemicals, such as α-Mangostin, and a plethora of studies have evaluated their health-promoting effects, including anti-metabolic [53], antioxidant [13], anti-inflammatory [11] and cardioprotective properties [57]. Despite mangosteen pericarp being one of the most important sources of natural phytochemicals, the yield of the phytochemicals and biological effects of the extracts is dependent on the extraction used. While several earlier studies have employed green extraction methods to valorize phytochemicals from mangosteen pericarp [9,14], our study explored another green extraction technique, PHWE, which only used water as an extraction solvent and featured simple instrumentation and procedures. In this study, we demonstrated that MPE using PHWE at 120 °C yielded a distinct phytochemical profile with potent antioxidant activity. Furthermore, using a global transcriptomic approach and pathway analysis, our results identified that the treatment of endothelial cells with 120 °C MPE upregulated key biological processes related to “steroid biosynthesis and metabolism”, likely involving the activation of the AMPK signaling pathway, suggesting the potential of valorizing mangosteen pericarp as a natural product to promote cardiometabolic health. As a result of the recent advancements and attractions in additive manufacturing techniques, other food products or food wastes, including mangosteen pericarp or MPE, may be explored in future studies for their use as food ingredients to create 3D-printed food for various healthcare applications [58]. 

## Figures and Tables

**Table 1:** Target compounds identified in mangosteen pericarp extract using LC/MSMS in negative mode.

No	Retention Time (Min)	Compound	Chemical Structure	Mass-to-Charge Ratio (*m*/*z*)	Tandem MS (MS/MS)
1	9.1	Gartanin	C_23_H_24_O_6_	395	395, 379, 351, 337, 325, 297, 285, 151, 124
2	9.1	α-Mangostin	C_24_H_26_O_6_	409	393, 377, 351, 339, 307, 295, 282, 267, 255, 242
3	8.5	γ- Mangostin	C_23_H_24_O_6_	395	395, 351, 339, 309, 297, 283, 271, 255, 242, 177

## Data Availability

No new data were created or analyzed in this study. Data sharing is not applicable to this article.

## Supplementary Materials

The following supporting information can be downloaded at: https://www.mdpi.com/article/10.3390/antiox12111932/s1, Supplementary Figure S1: MSMS spectra of (A) Gartanin, (B) α-mangostin and (C) g-mangostin from MPE. Supplementary Figure S2: List of DEGs obtained from ORA that were either upregulated (203 genes) or downregulated (205 genes) in HMEC-1 treated with MPE (0.05 mg/mL) for 48 h. Supplementary Figure S3: Upregulated DEGs associated with GO BP in HMEC-1 treated with MPE (0.05 mg/mL) for 48 h. Supplementary Figure S4: Downregulated DEGs associated with GO BP in HMEC-1 treated with MPE (0.05 mg/mL) for 48 h. Supplementary Figure S5: “AMPK signaling pathway” was an upregulated KEGG pathway for the transcriptomics profiling of HMEC-1 treated with MPE (0.05 mg/mL) for 48 h. Supplementary Table S1: RSD values (%) for peak heights of the respective analytes are based on the measurements at 254 nm (n = 5).
