# Supplementary material for: Pressurized Hot Water Extraction of Mangosteen Pericarp and Its Associated Molecular Signatures in Endothelial Cells
Source: Antioxidants (Basel). 2023 Oct 30;12(11):1932. doi: 10.3390/antiox12111932 (PMC10669822; doi:10.3390/antiox12111932)
Supplement: Supplementary file 1 [file antioxidants-12-01932-s001.zip › antioxidants-2625874-supplementary.pdf]

Supplementary Data

Pressurized hot water extraction of mangosteen pericarp and its associated molecular signatures in endothelial cells.

Sakeena Si Yu Tan[1, 3 *], Meyammai Shanmugham[2, *], Yu Ling Chin[2], Jia An[3], Chee Kai Chua[1,3], Eng Shi Ong[2, 3], Chen Huei Leo[2,3]

[1]Pillar of Engineering Product Development, [2]Science, Math & Technology, [3]Center for Healthcare Education, Entrepreneurship and Research (CHEERS), Singapore University of Technology & Design, Singapore 487372

*Equal contribution

Corresponding Author:

Dr Chen Huei Leo

Singapore University of Technology and Design,

8 Somapah Road, Singapore 487372, Republic of Singapore

E-mail address: chenhuei_leo@sutd.edu.sg

Tel.: +65 6434 8213

Supplementary Figures

Supplementary Figure S1

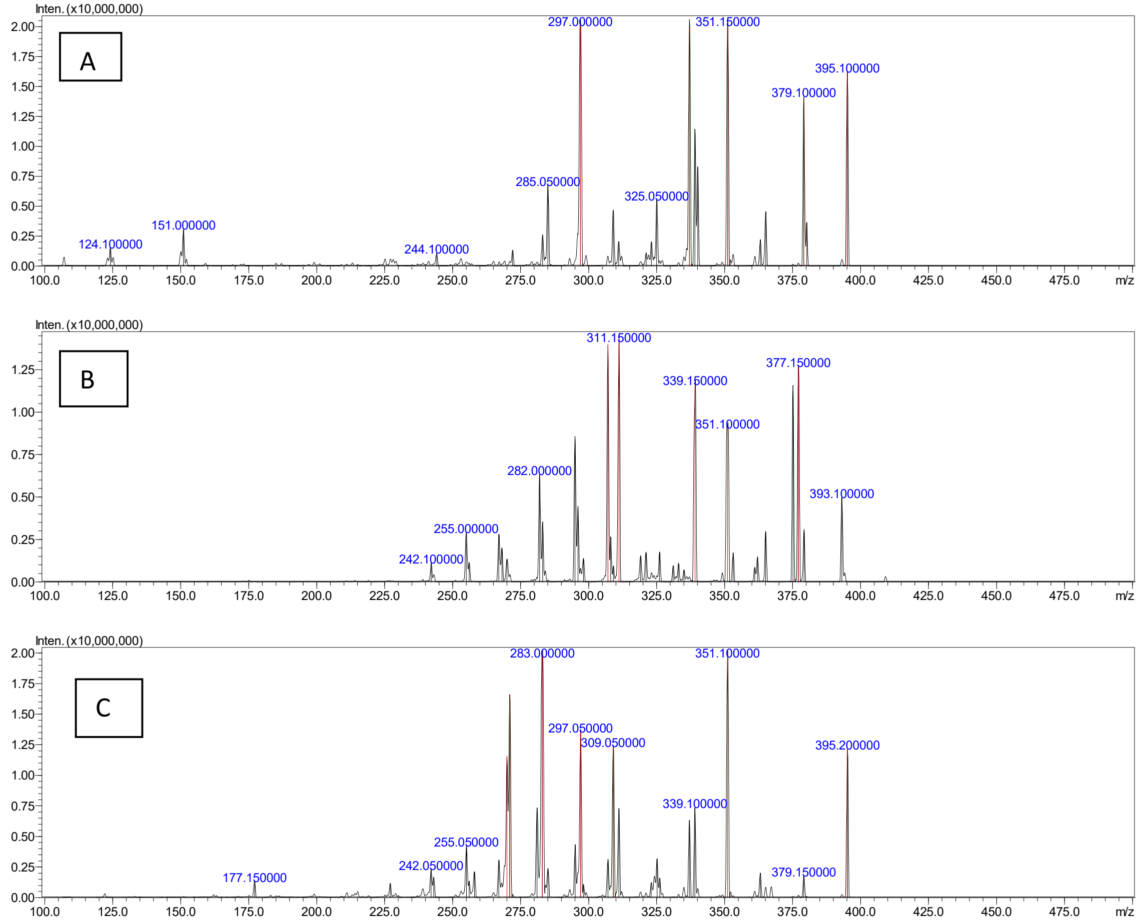

Supplementary Figure S1: MSMS spectra of A) Gartanin, B) α-mangostin and C) γ- mangostin from MPE

Supplementary Figure S2

Upregulated genes	Downregulated genes
FABP3, ANGPTL4, NPTX1, MALAT1, ANKRD36C, PCSK9, SFN, ZNF460, APOE, RAB3IL1, DHCR24, HMGCS1, SH2D5, DHCR7, FBLN5, SCD, FADS2, OSGIN1, PDK4, MSMO1, LSS, SYNE2, PLIN2, MVD, SQSTM1, INSIG1, FDFT1, ICAM1, NUPR1, TM7SF2, PTDSS2, NCR3LG1, CYP51A1, NSDHL, LDLR, FASN, SQLE, HMGCR, TMEM132D, SNX8, ITPKB, BHLHE40, MVK, HS1BP3, TRNL1, VAT1, RTN4R, IL32, ANKRD12, NOG, FZD5, SLC7A5, CTSD, TYMP, AKAP9, SERINC2, VEGFB, SLC15A3, KLF4, MMAB, RN7SL2, FTL, GNPDA1, LINC01537, MYORG, PLD6, VPS18, BMF, FDPS, SREBF2, COLEC12, RELB, UAP1L1, CSPG4, P2RY2, IRS2, CTNS, ACAT2, SLC2A6, TPP1, TRIM16, FADS1, SLC6A8, LIPA, RPP25, TBC1D2, DTX2, RRAGC, CD68, ZNF91, CITED4, HSD17B7, MX2, SLC38A7, PPP1R15A, PANK3, BLOC1S3, CYP27A1, NEDD4L, SLC25A1, SHISA2, SPNS1, GLMP, BLVRB, LPP, GRINA, ERG28, IDI1, DDIT4, FOXRED2, ZNF703, TRIB3, MGLL, ECH1, IER5L, NPC2, GYPC, TRPV2, ATP6V0D1, ITPRIP, FTH1, AKAP12, BRI3, CTSA, TP53I11, ORAI3, FN3K, SLC20A2, ND1, PCYT2, NRROS, CLN3, GRN, TRIM47, VAC14, LBX2-AS1, TGM2, TRAM2, JUND, CLIP2, TIPARP, HERC5, BRCA2, GOLGA4, BCL2L11, PHLDA3, CCDC69, ND2, ATP6V0C, SIPA1L3, MMP2, MCOLN1, DNASE2, TMEM97, MFGE8, ASS1, CPT1A, WBP2, MEGF9, WARS1, LRFN3, ARL4C, DUSP3, SNTA1, BOD1L1, ZFHX3, LHFPL2, SLC3A2, TKT, ATP6V1B2, SYNPO, PLEKHF1, HK1, KRT7, MAPKAPK3, ZNF385A, ATP6V0A1, ZNF628, MYH9, DAPK3, PPP1R3B, NUAK2, PSAP, PKN1, TOM1, LBH, SLC16A13, CTSS, CYSTM1, RAB32, RARA, CELSR1, SLC66A1, LRFN4, ATP8B1, MAFF, CLCN7, SH3BP4, PHYH, TOLLIP, PLEKHO1, HIC1, SPSB1	FGFR1OP2, CDH24, CLDN7, PSMD10, ZNF276, CTSC, RPL9, SORBS2, KAT2A, ASF1A, KATNBL1, MRPL13, PLPP5, RPS8, TAF1D, MRI1, HHEX, PAN2, ZNF302, CCDC59, ARRDC1-AS1, MNAT1, SDR39U1, MPZL2, CARD8 TRAPPC13, DGRL4, GNG11, SNRPG, MZF1, POLR2K, CCDC34, FKBP11, MAPKAPK5-AS1, SVIP, ABHD3, FAM219B, ADAMTS10, ESAM, NFKBIZ, BCKDHB, SLC35A1, MICALL2, SEMA6C, ADIRF, PIN4, CCNE2, SNHG8, CROCCP2, MBIP, RCBTB2, SNAP23, ARSJ, MTRF1, RAD51AP1, RCN2, CENPT, PDCD5, C20orf96, ZGRF1, LXN, ZCCHC9, C21orf58, FRG1HP, HAUS1, SMPD4BP, PNISR, TMEM80, CHTF18, GAS5, SDHAP4, MTERF2, HAUS5, NEK7, APBB3, PI4KAP2, CENPK, HERC2P3, TMA7, SFT2D1, WDR90 ARL6IP6, SELENOW, TMEM243, TESMIN, OTUD6B-AS1, REC8, ITGB3BP, UNKL, MRPL33, MYLIP, NUF2, PRPF39, SDHAP1, AASS, ARHGAP33, SNHG1, ERCC6L2-AS1, L3MBTL3, TTC14, CLK4, CDK10, CARD8-AS1, RBIS, KHDC4, LEPR, PET100, NALCN, FBXO43, RIDA, ZNF331, NDUFAF2, SLCO4A1, SOX18, GIMAP6, ENGASE, CDK5RAP3, ARHGAP27P1, FAM193B, CLEC2B, NDUFB1, STAG3L2, SNHG3, STAB1, ZNF692, KLHL3, GIMAP2, LOC102724023, LOC642846, NEIL1, SNHG5, NR2F1, PRICKLE4, LUC7L3, ARGLU1, PIF1, FAM216A, PGGHG, MYRIP, SFXN2, RHPN1, CREBZF, SH3D21, CSAD, WDR27, C6orf141, PFDN4, MTMR9LP, MIR503HG, CFAP119, STAG3L5P-PVRIG2P-PILRB, NOXA1, DDX12P, MATCAP1, ANXA2R, IFT20, TM4SF18, MGP, HOXD3, LINC00899, FRY, AMT, GMFG, NSUN5P2, SDHAP3, ZNF711, EMCN, MSTO2P, HOTAIRM1, ITGA10, SNHG10, SYCE1L, MIR3064, MIR5047, THUMPD3-AS1, RGL3, BRICD5, SNHG19, NSUN5P1, SEC31B, ARG2, CASP4LP, CCNL2, VAMP1, LINC01089, RSRP1, SPACA6, ID1, PAN3-AS1, PITPNA-AS1, LRRC70, MYCN, HOXD4, CAPS, CNTNAP3P2, LINC01311, MSMP, SNORD22, ABCA9, SNORD104 ABCG1, MAP3K2-DT, IZUMO4, SNORD50B, MOAP1

Supplementary Figure S2: List of DEGs obtained from ORA that were either upregulated (203 genes) or downregulated (205 genes) in HMEC-1 treated with MPE (0.05mg/ml) for 48 hours. DEGs were identified using p-value < 0.05, adjusted p-value < 0.05, and $\log_2 FC \geq = |0.4|$ criteria, n=3.

Supplementary Figure S3

GO Biological Process	Overlap	P-value	Adjusted P-value	Odds Ratio	Combined Score	Genes
Secondary alcohol biosynthetic process (GO:1902653)	18/34	1.26917E-27	2.07002E-24	119.6370968	7409.296973	IDI1;FDPS;MVK;HMGCS1;INSIG1;CYP51A1;MSMO1;DHCR24;HMGCR;HSD17B7;LSS;ACAT2;TM7SF2;SQLE;NSDHL;MVD;DHCR7;FDFT1
Cholesterol biosynthetic process (GO:0006695)	18/35	2.59006E-27	2.11219E-24	112.5939279	6892.788408	IDI1;FDPS;MVK;HMGCS1;INSIG1;CYP51A1;MSMO1;DHCR24;HMGCR;HSD17B7;LSS;ACAT2;TM7SF2;SQLE;NSDHL;MVD;DHCR7;FDFT1
Sterol biosynthetic process (GO:0016126)	18/38	1.86657E-26	1.01479E-23	95.69032258	5668.992198	IDI1;FDPS;MVK;HMGCS1;INSIG1;CYP51A1;MSMO1;DHCR24;HMGCR;HSD17B7;LSS;ACAT2;TM7SF2;SQLE;NSDHL;MVD;DHCR7;FDFT1
Cholesterol metabolic process (GO:0008203)	20/77	5.0975E-23	2.0785E-20	37.64111365	1932.144987	IDI1;FDPS;MVK;HMGCS1;INSIG1;CYP51A1;MSMO1;DHCR24;HMGCR;HSD17B7;LSS;ACAT2;TM7SF2;CYP27A1;SQLE;NSDHL;MVD;APOE;DHCR7;FDFT1
Regulation of lipid metabolic process (GO:0019216)	18/92	2.02443E-18	6.60368E-16	25.79163034	1050.783116	IDI1;FDPS;MVK;HMGCS1;CYP51A1;IRS2;HMGCR;LSS;SREBF2;TM7SF2;SQLE;NPC2;SCD;FASN;PSAP;MVD;DHCR7;FDFT1
Regulation of primary metabolic process (GO:0080090)	19/130	6.98452E-17	1.89863E-14	18.21353786	677.5481704	IDI1;FDPS;MVK;HMGCS1;CYP51A1;IRS2;HMGCR;LSS;SREBF2;TM7SF2;SQLE;SCD;FASN;PSAP;MVD;APOE;DHCR7;LDLR;FDFT1
Sterol metabolic process (GO:0016125)	11/70	1.21363E-10	2.82775E-08	19.06621586	435.3243575	CYP27A1;SQLE;NSDHL;INSIG1;CYP51A1;MSMO1;HMGCR;APOE;DHCR7;LIPA;TM7SF2
Steroid biosynthetic process (GO:0006694)	10/65	1.07387E-09	2.18936E-07	18.50140581	382.090922	CYP27A1;SQLE;INSIG1;CYP51A1;MSMO1;HMGCR;DHCR7;LSS;TM7SF2;FDFT1
Regulation of autophagy (GO:0010506)	15/231	1.59209E-08	2.88521E-06	7.194297472	129.1781839	CTSA;PLEKHF1;FZD5;DAPK3;SREBF2;RRAGC;PSAP;ATP6V1B2;SH3BP4;TRIB3;BMF;NUPR1;ATP6V0D1;ATP6V0C;ATP6V0A1
Lipid biosynthetic process (GO:0008610)	9/80	1.2424E-07	2.02635E-05	12.82231853	203.8883403	PCYT2;MVK;SCD;CYP51A1;MVD;HMGCR;LSS;FADS1;FDFT1

Supplementary Figure S3: Upregulated DEGs associated with GO BP in HMEC-1 treated with MPE (0.05mg/ml) for 48 hours. DEGs were identified using the p-value < 0.05, adjusted p-value < 0.05, and $\log_2 FC \geq = |0.4|$ criteria, n=3. The number of overlap genes in the pathway, odds ratio, combined score, p-value, adjusted p-value, and genes found in the respective GO BP are represented.

Supplementary Figure S4

GO Biological Process	Overlap	P-value	Adjusted P-value	Odds Ratio	Combined Score	Genes
DNA replication-independent nucleosome assembly (GO:0006336)	4/39	0.000690328	0.318639653	11.1234342	80.9601778	CENPT;CENPK;ASF1A;ITGB3BP
DNA-templated transcription, termination (GO:0006353)	5/70	0.000793623	0.318639653	7.512566641	53.63147673	TAF1D;MTERF2;SNRPG;MNAT1;POLR2K
regulation of cyclin-dependent protein serine/threonine kinase activity (GO:0000079)	5/82	0.001619074	0.34888331	6.337919506	40.72684088	PSMD10;HHEX;CCNE2;MNAT1;CDK5RAP3
positive regulation of gene expression, epigenetic (GO:0045815)	4/57	0.002868485	0.34888331	7.338972023	42.96213152	KAT2A;TAF1D;ASF1A;POLR2K
CENP-A containing chromatin organization (GO:0061641)	3/30	0.00361069	0.34888331	10.76579521	60.54528736	CENPT;CENPK;ITGB3BP
CENP-A containing nucleosome assembly (GO:0034080)	3/30	0.00361069	0.34888331	10.76579521	60.54528736	CENPT;CENPK;ITGB3BP
termination of RNA polymerase I transcription (GO:0006363)	3/30	0.00361069	0.34888331	10.76579521	60.54528736	TAF1D;MNAT1;POLR2K
mRNA splice site selection (GO:0006376)	3/30	0.00361069	0.34888331	10.76579521	60.54528736	PRPF39;LUC7L3;KHDC4
chromatin remodeling at centromere (GO:0031055)	3/32	0.004344749	0.34888331	10.02231237	54.50922615	CENPT;CENPK;ITGB3BP

Supplementary Figure S4: Downregulated DEGs associated with GO BP in HMEC-1 treated with MPE (0.05mg/ml) for 48 hours. DEGs were identified using the p-value < 0.05, adjusted p-value < 0.05, and $\log_2 FC \geq = |0.4|$ criteria, n=3. The number of overlap genes in the pathway, odds ratio, combined score, p-value, adjusted p-value, and genes found in the respective GO BP are represented.

Supplementary Figure S5

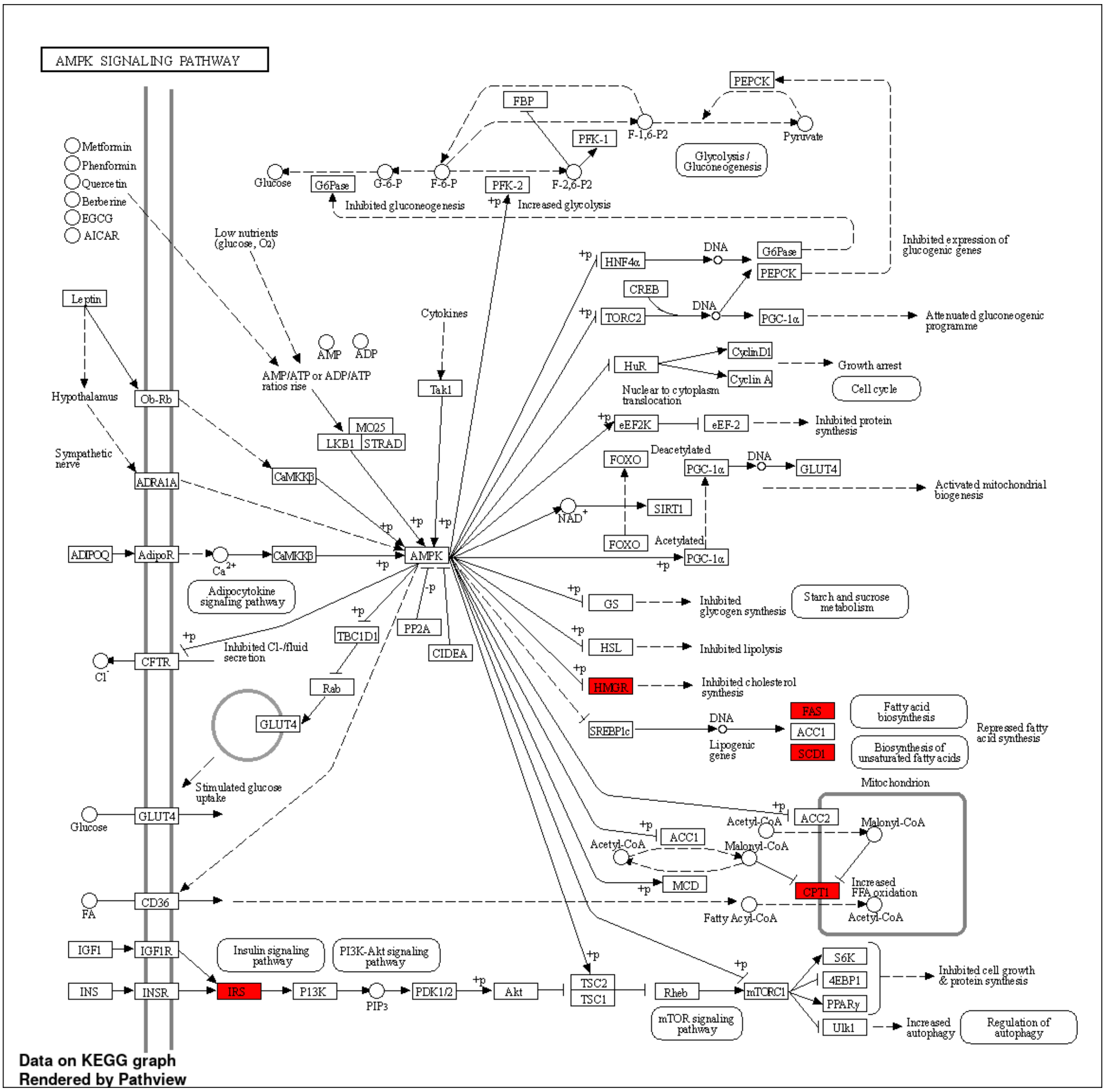

Supplementary Figure S5: "AMPK signalling pathway" was an upregulated KEGG pathway for the transcriptomics profiling of HMEC-1 treated with MPE (0.05mg/ml) for 48 hours. Gene hits are highlighted in red (upregulated). DEG was identified using adjusted p-value < 0.05 and $log_2FC \geq = |0.4|$ criteria, n=3.

Supplementary Tables

Supplementary Table S1

**Compound**	**Calibration Curve (Range: 0 – 100 mg/L)**	**R^2**	**Relative Standard Deviation (RSD) (%) injection repeatability conducted on 8mg/L**
**α-Mangostin**	y = 77791x - 17816	0.9891	0.64
**γ-Mangostin**	y = 166541x - 43050	0.9984	0.77
**Gartanin**	y = 105297x – 8755.8	0.9965	0.54

RSD values (%) for peak heights of the respective analytes are based on the measurements at 254 nm (n= 5).
